# Memory bias and personality characteristics in college students with social anxiety disorder

**DOI:** 10.47626/2237-6089-2020-0042

**Published:** 2022-09-13

**Authors:** Carmem Beatriz Neufeld, Priscila Goergen Brust-Renck, Priscila de Camargo Palma, José Alexandre de Souza Crippa

**Affiliations:** 1 Departamento de Psicologia Faculdade de Filosofia Ciências e Letras de Ribeirão Preto Universidade de São Paulo Ribeirão Preto SP Brazil Departamento de Psicologia, Faculdade de Filosofia Ciências e Letras de Ribeirão Preto, Universidade de São Paulo (USP), Ribeirão Preto, SP, Brazil.; 2 Programa de Pós-Graduação em Psicologia Universidade do Vale do Rio dos Sinos São Leopoldo RS Brazil Programa de Pós-Graduação em Psicologia, Universidade do Vale do Rio dos Sinos (UNISINOS), São Leopoldo, RS, Brazil.; 3 Departamento de Neurociências e Ciências do Comportamento Faculdade de Medicina de Ribeirão Preto USP Ribeirão Preto SP Brazil Departamento de Neurociências e Ciências do Comportamento, Faculdade de Medicina de Ribeirão Preto, USP, Ribeirão Preto, SP, Brazil.

**Keywords:** Personality, social phobia, memory, emotions, arousal

## Abstract

**Introduction:**

Some individuals are more susceptible to recalling false information about events that never happened in their life. Nevertheless, there are several factors, such as personality characteristics, that appear to be related to memory performance. Social anxiety also provokes memory deficits for events that happen to other people, because these individuals tend to focus on their own inner selves rather than on external signs.

**Objective:**

To investigate the influence of the personality characteristics of individuals with social anxiety disorder (SAD) on memory performance.

**Methods:**

In this study, 183 university students had their memory tested using a complex emotional story about a mother and her son. Only subjects without clinical symptoms of depression and general anxiety (N = 148; 61 with SAD) were included in the study. Participants were compared for differences in personality characteristics using the Factorial Inventory of Personality and for SAD using the Social Phobia Inventory.

**Results:**

The main results showed that memory performance of individuals with low percentile ranks in the personality characteristic dominance, i.e., those with low self-esteem, remembered more true information about the story than those with high scores when they did not have SAD.

**Conclusion:**

The results are helpful to foster better understanding of the personality characteristics related to SAD, such as low dominance, which implies low self-esteem and difficulties with trust and with imposing themselves on others. The results could help development and improvement of techniques for therapeutic intervention.

## Introduction

Human memory is influenced by several factors, such as prior knowledge, current mental state, and emotions.^1 - 3^ A large part of these differences occur because of individual dissimilarities, particularly in intelligence and personality characteristics.^4 , 5^ Some individuals seem to be more susceptible to variations affecting memory performance, especially with regard to distortions such as recalling false memories.^1^ Susceptibility to forming false memories (recalling events that never occurred, or that occurred in a different way from how they were retrieved) may result in serious consequences, directly affecting individuals’ lives. For example, misremembering what happened to other people during an accident (e.g., how severely injured they were, what part of the body was injured) due to focusing on inner wounds or flashbacks from memories of traumatic accidents.

The fact that some individuals are more prone than others to recall false information about events that never happened in their life prompts us to question what we know regarding the role individual differences play.^3 , 4^ Current research has failed to reach a consensus on which types of individuals are more susceptible or resistant to false memories (e.g., those with high levels of conscientiousness, neuroticism, perfectionism, etc.).^4^ Some researchers claim that certain personality characteristics lead to a tendency to memory failures^6^ and others suggest that it is difficult to detect which individual differences are related to increased false memories.^7^ According to Payne et al.,^8^ individuals with high stress levels were more likely to remember false information. However, Beato et al.^7^ found that acute stress was not related to false memory susceptibility.

Nevertheless, according to the cognitive model, distorted or dysfunctional thoughts are one of the characteristics common to all psychological disorders. According to this model, behavior and thought patterns are responses shaped by environmental, intrapersonal, interpersonal, or biological interactions, all of which influence personality characteristics. This model suggests that perhaps there is no single factor that influences memory performance, but instead a set of factors intervene in the development of true and false memories. For example, patients with high rates of delusion are less likely to remember information about an event and more likely to produce false memories.^9 , 10^

People with anxiety disorders produce unique results regarding memory performance. Socially anxious individuals tend to examine their own inner selves, finding it difficult to devote their attention to external signs, generating memory deficits.^11^ Liang et al.^12^ showed that individuals with social anxiety forgot words with a positive emotional content more easily than healthy individuals. Along the same lines, Toffalini et al.^13^ showed an increase in false memory creation for negative events (but not for positive ones), in individuals with high levels of anxiety, even when controlling for other disorders (e.g., depression).

However, this is not always the case, and some researchers suggest that anxiety in and of itself does not affect memory.^1 , 3^ Neufeld et al.^14^ have addressed this issue, suggesting that personality characteristics along with psychological disorders (i.e., anxiety) were related to memory distortion. They tested the memory performance of 200 university students to study possible effects of personality characteristics on mnemonic distortion of word lists. The results showed that social desirability was one of the predictors of memory performance in this population, a notable characteristic in individuals with social anxiety, which has a prevalence of 6.8% in the adult population.^15^

In this study, we evaluated differences in memory performance between individuals with and without social anxiety disorder (SAD) with different personality characteristics. Although several studies have investigated these factors’ independent associations with memory,^4 - 7^ few have combined memory for complex memory performance, individual differences, and SAD. However, these studies (e.g., Neufeld et al.^14^ ) did not test memories of complex emotional events. According to the APA,^16^ SAD is characterized by an intense fear of social situations or situations in which one’s achievement may be negatively evaluated and subjected to criticism, fostering hypersensitivity to criticism or even to negative assessments from others, and involving low self-esteem. The fear of negative evaluation may also result in avoidance and safety behaviors, causing criticism to go unanswered^17^ and lack of positivity and negative cognitions have been associated with fear and avoidance in social anxiety disorder.^18^ One of the factors that may contribute to this phenomenon is the social cost involved in the act of disagreeing, which is correlated to the fear of negative evaluation and possible social isolation.^19^ Lifetime prevalence of all anxiety disorders in Brazil was estimated to be around 10-28% and the prevalence rate was about 27.5% for the age group from 18 to 34 years old,^20^ which is consistent with the college-aged participants in the present study.

Taking the literature into consideration and based on the concept of automatic thoughts that lead to cognitive errors described in the Cognitive Behavioral Therapy model,^21^ our hypothesis is that individuals whose thoughts are highly independent and rigid and who interpret social relationships more as a source of deprivation than of pleasure (e.g., people with social phobia and personality characteristics with high dominance, high autonomy, and low change) might show a tendency toward greater distortion of information with negative emotional content, in particular when tested for information that simulates real life events, such as Neufeld et al.’s Slide Presentation Procedure, which targets negative emotional content.^22 , 23^ We also tested the hypothesis that individuals with extreme variants of normal personality characteristics would be more susceptible to mnemonic distortions for emotional events^14 , 24^ and that individuals with SAD should also be more prone to answering the questionnaire on individual differences in a manner considered more socially acceptable (higher social desirability), which might contribute to discrepant mnemonic results. Finally, memory performance of individuals with SAD should be more susceptible to errors for individuals whose personality characteristics are associated with social disorders. Dysfunctional personality characteristics are often associated with clinically important psychosocial functioning. Marteinsdottir et al.^25^ identified that individuals with SAD were more likely to show impaired socialization skills.

## Method

### Design

Regarding the memory variables, the present study employed a full factorial mixed, quasi-experimental 2x3x3 design, with repeated measures for the last two variables. The first independent variable was the emotional version of the story (emotional arousal or non-arousal); both versions had negative valence. Participants were randomly assigned to 1 of the 2 versions to ensure that between-subject variables were not liable to selection bias or possible diagnostic confounds, as well as to meet the necessary assumptions for data analysis. The second and third independent variables were story phase and item of memory test. All participants received the target material divided into three phases. The three phases were separated for the purposes of analysis, even though they are all part of a sequential uninterrupted narrative. Phases 1 and 3 are the same for both versions of the story and phase 2 presents a different narrative for each version based on arousal levels. Three types of items were assessed in the memory test (target items, related distractors, and non-related distractors) based on the story content. The dependent variable was memory performance in the recognition test for an ecologically valid event that simulates real life.

Memory data were analyzed separately based on participants’ characteristics: first, we compared the performance of individuals with low and high levels of social desirability (need to please the researcher) to identify those who might not have been truthful in their answers to personality tests (and exclude them from further analyses); then, we compared differences between people with low and high levels of each personality characteristic (above the 75% quartile and below the 25% quartile); finally, we investigated how SAD affected individuals with different levels of each personality characteristics (participants were clinically evaluated for presence and absence of social anxiety).

### Participants

One hundred and eighty-three university students from Ribeirão Preto, São Paulo, Brazil took part in the study. Participants were between 17 and 34 years old (mean age = 22.20, standard deviation [SD] = 3.54) and 65% were female. They were recruited by convenience and snowball sampling at the University. The inclusion criteria were as follows: no symptoms of depression or anxiety. Overall, 148 participants comprised the sample, 61 of whom had SAD and 64% of whom were female. From these, 34 were randomly assigned to watch the arousal version of the story (mean age = 22.99, SD = 3.83) and 27 to watch the non-arousal version (mean age = 21.59, SD = 3.93). There were 87 participants without SAD, 59% of whom were female. From these, 55 were randomly assigned to watch the arousal version of the story (mean age = 23.22, SD = 3.88) and 32 to watch the non-arousal version (mean age = 21.34, SD = 2.88).

### Instruments

Clinically relevant depression was evaluated with the Brazilian version^26^ of the Patient Health Questionnaire-9 (PHQ-9)^27^ (Cronbach’s = 0.53) and the Brazilian version^28^ of the Beck Depression Inventory (BDI)^29^ (Cronbach’s = 0.81). Scores of six or less on the PHQ-9 and 10 or less on the BDI were considered indicative of no significant symptoms of depression. In addition, participants were included who scored seven or less on the Brazilian version^30^ of the Self-Report Questionnaire (SRQ-20)^31^ (Cronbach’s = 0.72), indicating quality of life and well-being.

Individuals with SAD were identified by scores greater than or equal to 19 on the Brazilian version^32^ of the Social Phobia Inventory (SPIN)^33^ (Cronbach’s = 0.93), indicating presence of symptoms that are compatible with a diagnosis of SAD, and by scores greater than or equal to 20 on the Brazilian version^28^ of the Beck Anxiety Inventory (BAI)^34^ (Cronbach’s = 0.90), which indicates the severity of anxious symptoms; moderate symptoms range from 20 to 30 points, and severe symptoms range from 31 to 63.^28^ Patients with clinically elevated scores for SAD according to the SPIN and BAI measures also underwent the Brazilian version^35^ of the anxiety module of the Structured Clinical Interview for DSM-IV, clinical version (SCID-CV),^36^ to confirm the diagnosis and verify absence of comorbidities.

Participants’ memory was evaluated using the Slide Presentation Procedure.^22 , 23 , 37^ The material was composed of a sequence of 11 images accompanied by two versions of a narrative: control and experimental (arousal). The story was divided into three phases: phases 1 and 3 were identical for both groups, and phase 2 was a critical phase differentiating between arousal and non-arousal versions based on whether characters were involved in an accident or just witnessed an accident happening. Phases 1 and 3 are equal in terms of content (pictures and narrative). Both versions present negative emotional content.

We conducted a memory test^38^ with 84 recognition items: 42 target items that corresponded to measures of true memories (i.e., exactly what was shown in the story), 30 distractors to measure false memories (i.e., items consistent with the story content, but which were not presented), and 12 unrelated distractors to assess non-mnemonic answers (intrusions) resulting from mistakes or guessing. For each item, participants were asked to mark the option “yes” when the sentence corresponded exactly to the information that was presented in the target material or “no” for all information that was not seen or heard, even if such information could have been inferred about the event. Results were averaged, and higher scores indicate superior performance on each subscale.

To evaluate personality characteristics, we used the 145-question Factorial Inventory of Personality (FIP),^39^ which evaluateds14 distinct characteristics, controlled for social desirability (12 items) and instrument validity (8 items) to confirm the reliability of the answers obtained from the inventory. The theoretical basis for FIP assessment is Edwards’ Personal Preference Schedule. For each question, participants were asked to mark the extent to which that item represented a characteristic of their personality on a Likert scale, from 1 (not characteristic) to 7 (totally characteristic). The personality characteristics refer to nurturance (desire to assist helpless people or people who are in a needy situation), intraception (allowing oneself to be led by feelings and widespread inclinations, being dominated by the pursuit of happiness, by fantasies and imagination; besides judging others on their real or alleged intentions, and not really on the actions themselves), succorance (need to receive affection from cherished people), deference (admiring and supporting a superior person), affiliation (taking pleasure in contributing with an ally), dominance (controlling the people in one’s environment), denegation (accepting criticism without reacting), achievement (receiving recognition for executed tasks), exhibition (desire to thrill, impress or entertain others), aggression (overcoming opposition by force or disparaging others), order (need for precision, organization, and cleanliness), endurance (ability to complete projects), change (taking delight in what is different, new or exotic), and autonomy (breaking free from constraints, going by one’s own rules). Responses are evaluated based on the percentile ranks of each personality factor assessed: scores below 25 are considered “extremely low” and those above 75 are considered “extremely high.”

### Procedure

After approval by the Institutional Review Board (CEP-FFCLRP #534/2010), data were collected in groups in quiet rooms. All the steps of the study were explained and the volunteers were assured that they were free to stop and leave the study at any time, after which each participant signed the Informed Consent Form, prepared according to the ethical guidelines that regulate research involving human beings. All participants completed the entire procedure.

Initially, participants were instructed to pay close attention to the target material and especially to avoid making comments throughout the procedure. The material was presented using multimedia equipment. Next, the FIP was administered to participants, lasting on average 40 minutes, during which participants were instructed to read the sentences of the inventory and mark after each sentence how much it described their personality, ranging from “not at all characteristic” to “totally characteristic.” Finally, they took the recognition memory test.

Data were analyzed using IBM SPSS Statistics, version 21. We used repeated measures Analysis of Variance (ANOVA) to analyze the data obtained from these instruments. All statistical treatments adopted α = 0.05 for hypothesis tests. Post hoc comparisons were carried out with Bonferroni correction. All measures were normally distributed. Results concerning the relationships between clinical variables and personality characteristics are beyond the scope of this paper and are available as supplementary material.

## Results

Overall effects of memory performance were analyzed by means of a 2x3x3 ANOVA with repeated measures for story version, phase, and type of item. Results showed a main effect of memory [ *F* (2,125) = 1907.59, p < 0.001, = 0.968], with higher rates of true memory (M = 0.74, SD = 0.11) than false memory (M = 0.37, SD = 0.13), both of which had higher rates than intrusions (M = 0.03, SD = 0.06; p < 0.001). This confirms appropriate recognition rates for true and false memory for the type of materials used – for comparison, see Neufeld et al.^14^ and Palma et al.^40^ Rates of non-mnemonic responses (or guesses) were very low, suggesting that participants were paying attention to the task.

### Memory differences due to social desirability

The effect of trying to please the researcher was investigated in a 2x3x2 ANOVA for each version of the story with repeated measures for type of information recovered and high and low levels of social desirability. The results showed significant interactions among the three factors [ *F* (2,61) = 5.83, p < 0.05, = 0.161]. Post hoc comparisons showed that this effect was due to true memory performance (evaluated as acceptance of target items), which was higher for participants with low social desirability (M = 0.79, SD = 0.07) than for those with high social desirability (M = 0.68, SD = 0.15) in the arousal version of the story (p < 0.05). Furthermore, among participants with high social desirability, those who watched the arousal version of the story recalled less true information than those who watched the non-arousal version (M = 0.78, SD = 0.10).

In order to control for desire to please the researcher (which was not the goal of the present study) and obtain more direct data from the actual personality differences, participants with high social desirability indexes were excluded from the analyses (index percentage greater than or equal to 75). In total, 35 participants were excluded. The sample used in the remaining analyses was divided as follows: among participants without SAD, 32 watched the arousal version (M = 21.63 SD = 2.79) and 24 watched the non-arousal version (M = 20.87, SD = 2.31), and among participants with SAD, 17 watched the arousal version (M = 20.24, SD = 2.20) and 19 watched the non-arousal version (M = 21.05, SD = 4.30). No significant differences were found between groups (SAD by story version) in terms of age and sex of the participant (ps > 0.05).

### Memory differences due to personality characteristics

The FIP personality characteristics were categorized according to the degree of the characteristic that one piece of information represents, and only evaluated at levels that were extremely low (5 to 25 percentile) or extremely high (75 to 100 percentile) for the entire sample. This division was employed so that only participants with very pronounced levels of some personality characteristics were observed, since, according to the test rules, the intermediate levels of the characteristics represent a personality functioning typically. All participants except one presented at least one characteristic at an extreme level. To investigate the influence of the personality characteristics on each type of memory, a 2x3x2 ANOVA was conducted for each personality characteristic (at its two extreme levels) with repeated measures story phase and version, controlling for high social desirability. The results for personality characteristics that showed an interaction are presented in Table 1 (controlling for social desirability).

**Table 1:** Memory performance for personality characteristics that showed an interaction with story phase

	Phase 1	Phase 2	Phase 3
True memories			
Low autonomy	0.72 (0.13)	0.82 (0.13)*	0.65 (0.15)
High autonomy	0.79 (0.14)*	0.78 (0.14)*	0.66 (0.13)
False memories			
Low affiliation	0.29 (0.11)	0.23 (0.14)	0.56 (0.15)*
High affiliation	0.43 (0.13)*	0.23 (0.14)	0.49 (0.21)*
Low intraception	0.36 (0.14)	0.24 (0.14)	0.56 (0.24)*
High intraception	0.47 (0.16)*	0.24 (0.14)	0.50 (0.22)*

Data presented as mean (standard deviation).

* p < 0.05.

Our analyses of true memory recognition indicated a significant interaction between story phase and the personality characteristic autonomy [ *F* (2,45) = 3.32, p < 0.05, = 0.129]. Results showed that individuals with low levels of autonomy recalled more true information from phase 2 than from phase 1 (p < 0.05). In other words, dependent individuals (i.e., low autonomy) seem to be more involved with the task and recall more information that is correct about the critical phase of the story. Phase 3 (post-emotional event) does not present new information and seems to be irrelevant in either case, and true memory performance was lower than for phase 2 (p < 0.001) for those with low autonomy. However, recall of phase 1 was greater for those with high autonomy in comparison to those with low autonomy (p < 0.05). In other words, individuals who are not concerned about others, i.e., who are more autonomous (i.e., high autonomy) possibly recall more because they do not need to worry about others and are paying attention to the stimulus. These participants with high autonomy are more likely to remember true information from both phases 1 and 2 when compared to phase 3, after the negative content and emotional arousal are introduced in the storyline (ps < 0.001).

The analyses of false memories show an interaction of story phase with the personality characteristic affiliation [ *F* (2,49) = 5.62, p < 0.01, = 0.186]. Participants with low levels of this characteristic were more likely to produce more false memories for phase 3 than 1 (p < 0.001) and those with high levels of the characteristic affiliation showed greater production of false memories for phase 1 than for phase 2 (p < 0.001). Overall, participants with both low and high affiliation were more likely to produce false information for phase 3 than phase 2 (ps < 0.001). These results suggest that independent participants (i.e., low affiliation) were susceptible to mnemonic distortions to story information after the crucial phase of the story (i.e., phase 2 in which the negative content was included). Participants who were dependent on the group (i.e., high affiliation), however, seemed to produce the same level of false memories from both neutral phases (i.e., 1 and 3). The presence of emotion in phase 2 of the story seems to have granted even higher protection (i.e., lower production of false memories in comparison to both other phases) to participants who feel pleasure in contributing to the group (i.e., high affiliation).

False memory production also had significant interaction between phase and the personality characteristic intraception [ *F* (2,33) = 3.83, p < 0.05, = 0.189]. Results showed that individuals who focus more on the actions per se (i.e., low intraception) produced more false memories for phase 3 than for phase 1 (p < 0.01) and phase 2 (p < 0.001). Those who allow themselves to be led by feelings (i.e., high intraception) were more susceptible to mnemonic distortions in the two neutral phases of the story (i.e., 1 and 3) compared to the critical phase 2 (ps < 0.001). Again, individuals with high levels of the personality factor (in this case, intraception) seem to have been protected against the production of false memories for the non-emotional phase of the story.

### Interaction between personality characteristics, social anxiety, and memory performance

To investigate the interaction between SAD and personality characteristics, a 2x2x3 ANOVA was conducted for each personality characteristic at its two extremes for both story versions, by type of information recovered ( Table 2 ). Only the results of the personality characteristics that showed an interaction are presented. The analyses show significant interactions between SAD and the personality characteristic dominance [ *F* (2,46) = 3.01, p < 0.05, = 0.116] for true and false memory performance. In this case, individuals without SAD were more likely to retrieve true information than those with SAD (p < 0.05) when they were low in dominance (i.e., had higher feelings of insecurity, low self-esteem). Individuals low in dominance have difficulty imposing themselves in groups and controlling their environment. Overall, those without SAD remembered more true information if they were in the low percentile rank of the personality characteristic dominance compared to those in the high percentile (p < 0.05). Moreover, those with SAD showed a tendency to produce more false memories than those without SAD (p < 0.05) when they show feelings of confidence and have a desire to control others (i.e., have high dominance).

**Table 2:** Memory performance by symptoms of social anxiety disorder and levels of the personality characteristic dominance

	Without SAD		With SAD	

	Low	High	Low	High
True memories	0.80 (0.07)*	0.72 (0.10)	0.70 (0.10)	0.75 (0.10)
False memories	0.39 (0.15)	0.34 (0.12)	0.35 (0.09)	0.42 (0.11)*
Non-mnemonic responses	0.02 (0.04)	0.01 (0.03)	0.05 (0.09)	0.02 (0.04)

Data presented as mean (standard deviation).

* p < 0.05.

## Discussion

The main goal of the study was to investigate the influence of personality characteristics (and to ascertain which characteristics play a role) on the susceptibility to memory distortions of individuals with and without SAD. Overall, results show that individuals with SAD and specific personality characteristic were susceptible to mnemonic challenges. These findings contribute to the understanding of how personality characteristics and psychological symptoms, such as SAD, affect memory performance. The results are discussed in order in which they were presented.

It was shown that the characteristic autonomy showed a significant interaction with true memories. Autonomy is related to feeling free, acting independently, and following one’s own impulses, without being subservient to others. Both participants with high and low autonomy scores had higher true memory indexes for the emotional phase of the story, however, those with high autonomy were also more likely to remember the beginning of the story. That is, people with little autonomy who are more dependent seem to recall more information that is true from the emotional arousing phase than from the two neutral phases, or even to pay more attention to the story. This result is in line with empirical data from participants from the same country,^24^ since individuals with a chronic level of emotional misalignment and instability (i.e., those who are insecure and inadequate) seem to be more impressionable and more susceptible to distortions. Participants with high autonomy worry less about others and stay more attentive to stimuli. According to Wright et al.,^19^ people who are less self-confident accept the way the situation was recalled by the more self-confident person. Evidence was found that participants who initiate the conversation, or show more indications of autonomy, are less easily influenced. This was more evident in phase 1 than in the other phases, that is, the rates of true memories related to autonomy were higher at the beginning of the story.

False memories were associated with the characteristic affiliation. Showing the importance of affective characteristics, such as giving and receiving affection, high affiliation indicates the need to maintain affective bonds and demonstrate affection. The overall production of false memories for the characteristic affiliation was higher in phase 3, that is, in the last phase, immediately after the emotional content and arousal information were presented. Participants with low affiliation are more independent and less sensitive to the bonds in the story, which explains how they were more susceptible to interference after the critical phase, suggesting they were disturbed by the storyline and produced more false memories after the fact. Those with high affiliation were equally likely to produce false memories for both neutral phases of the story, but not for the critical phase. The literature has reported a protective effect of emotion on memory and suggested that emotional events were recalled in greater number.^12 , 14 , 18 , 23^

The relationship between SAD and the FIP personality characteristics was also evaluated against memory performance. Participants without SAD presented a higher true memory index when associated with the characteristic dominance, having, however, low dominance scores. This characteristic is related to an expression of feelings of self-confidence and to the desire to control others. Notwithstanding, participants who recalled more true information presented low dominance. Although the analysis of SAD and the personality characteristics measured by the FIP did not indicate a correlation between SAD and dominance, this characteristic seems to have an impact on recalling true memories, i.e., participants who do not desire to control their environment might be more attentive to the mnemonic task. In other words, individuals with SAD who also have difficulty in imposing themselves within the group and controlling their environment (i.e., low dominance), were impaired in recalling the facts when compared to those without SAD but with the same personality characteristics. These results suggest a clear distinction between a feeling of inadequacy regarding one’s environment that is a classic sign of an individual with SAD and the presence of the disorder when it comes to the information of events recovered.^40 , 41^

Thus, the results of this study are helpful to foster better understanding of the personality characteristics related to SAD and also provide evidence on how personality characteristics may impact the memory process of this population, which could help development and improvement of techniques for therapeutic intervention. Finally, we can clearly state that some personality characteristics impact mnemonic performance. However, studies with larger samples, assessed in different manners, not through self-report, will improve the generalizability of the results. Future studies should also consider ways to control SAD levels and use different instruments for measuring personality in order to consolidate the results.

## Supplementary material


