# Supplementary material for: Memory bias and personality characteristics in college students with social anxiety disorder
Source: Trends Psychiatry Psychother. 2022 Sep 13;44:e20200042. doi: 10.47626/2237-6089-2020-0042 (PMC10039717; doi:10.47626/2237-6089-2020-0042)
Supplement: Supplementary file 1 [file 2238-0019-trends-44-e20200042_suppl01.pdf]

http://doi.org/10.47626/2237-6089-2020-0042

Supplementary Material: Additional Results

Relationship between clinical variables and personality characteristics

Social phobia and personality

A correlation analysis was conducted to assess the relationships between social phobia and the different personality characteristics evaluated by the FIP. As was expected, a correlation was observed between social phobia and the denegation characteristic (r_s = 0.32, $p < 0.001$), showing that participants with higher indexes of social phobia tend to accept more criticism without reacting. Participants with higher indexes of social phobia also take less delight in what is different, new, or exotic (based on the change characteristic; r_s = -0.24, $p < 0.05$). On the other hand, participants with higher indexes of social phobia tend to be worse at overcoming opposition by way of force or disparaging others (based on the aggression characteristic; r_s = 0.25, $p < 0.05$).

A multiple regression analysis was conducted using the level of social phobia as the dependent variable and the personality characteristics as the independent measure, revealing an effect of the characteristics succorance, change, and denegation (R^2 = 0.28, F(14, 145) = 5.18, $p < 0.001$). The denegation variable had the largest standard angular coefficient, showing that it explains the greatest amount of variance (β = 0.40, t(145) = 4.88, $p < 0.001$). The positive sign of the coefficient shows that higher scores of denegation were associated with higher levels of social phobia. In other words, participants with more social phobia accept more criticism without reacting. High levels of the succorance characteristic were also associated with more social phobia (β = 0.25, t(145) = 2.58, $p < 0.05$), indicating that participants with social phobia need to receive affection from people dear to them. Finally, the negative sign of the coefficient for the change characteristic (β = -0.29, t(145) = -3.30, $p < 0.05$) shows that participants with more social phobia are more averse to what is different and new. This result is consistent with the feeling of inadequacy that characterizes social phobia disorder. It is worth noting that when people with social phobia take a stand, they are more aggressive (even though aggression is not predictive of social phobia when controlled for other personality characteristics).

Other clinical characteristics and personality

Regarding the other clinical characteristics, correlations were observed with BAI indexes, which evaluate characteristics of general anxiety, and of personality. Specifically, high BAI indexes were related to increased need to receive affection (succorance; r_s = 0.22, $p < 0.05$), to obtain recognition (achievement; r_s = 0.20, $p < 0.05$), and to overcome opposition by force (aggression; r_s = 0.25, $p < 0.05$), as well as to a greater tendency to accept criticism without reacting (denegation; r_s = 0.24, $p < 0.05$).

A multiple regression analysis was conducted using the level of anxiety as the dependent variable and the personality characteristics as the independent measure, revealing only an effect of the characteristics succorance and denegation (R2 = 0.21, F(14, 145) = 3.78, p < 0.001), indicating that these characteristics are predictive of anxiety even when controlled for other

characteristics. The succorance variable had the largest standard angular coefficient, showing that it explains the greatest amount of variance ($\beta = 0.33$, $t(145) = 3.23$, $p < 0.05$). The positive sign of the coefficient shows that participants with high levels of anxiety feel a greater need to receive affection from people dear to them. High levels of anxiety were also associated with denegation ($\beta = 0.30$, $t(145) = 3.53$, $p < 0.05$), showing that participants with higher anxiety accept criticism without reacting. As a group, this result suggests that anxiety is, above all, related to dependence on others, observed by way of seeking affection and accepting criticism. (Note that aggression was not significant when controlling for other characteristics, but a certain level of aggression is consistent with high levels of anxiety in specific situations.)

Regarding associations with PHQ-9 and BDI results, which assess depression symptoms, both were shown to be associated with personality characteristics, in that the more characteristics associated with depression, the greater the need to overcome opposition by force (aggression; $r_s = 0.30$ e $r_s = 0.28$, $ps < 0.05$, respectively) and the greater the tendency to accept criticism without reacting (denegation; $r_s = 0.29$ e $r_s = 0.22$, $ps < 0.05$, respectively). A multiple regression analysis was conducted using level of depression on either the PHQ-9 or the BDI as the dependent variable and the personality characteristics as the independent measure, revealing an effect of the characteristic denegation ($R^2 = 0.24$, $F(14, 145) = 4.43$, $p < 0.001$ e $R^2 = 0.22$, $F(14, 145) = 3.93$, $p < 0.001$, respectively), indicating that participants with high levels of anxiety have a greater need to receive affection from people dear to them when controlling for other personality characteristics ($\beta = 0.46$, $t(145) = 5.53$, $p < 0.001$ e $\beta = 0.40$, $t(145) = 4.69$, $p < 0.001$, respectively). Furthermore, the nurturance characteristic was also predictive of depression, according to the PHQ-9 ($\beta = -0.32$, $t(145) = -2.54$, $p < 0.05$). The negative sign of the coefficient indicates that people with high levels of depression do not show a desire to help others who are in need, which is consistent with the feeling of depression.

Finally, high indexes on the quality-of-life inventory were associated with a greater tendency to admire a superior and accept criticism without reacting (denegation; $r_s = 0.21$, $p < 0.05$). A multiple regression analysis was conducted using quality of life as the dependent variable and the personality characteristics as the independent measure, also revealing an effect of the characteristic denegation ($R^2 = 0.22$, $F(14, 145) = 3.95$, $p < 0.001$) when controlling for the other variables ($\beta = 0.40$, $t(145) = 4.71$, $p < 0.001$). Although quality of life is related to the tendency to accept criticism without reacting, the succorance characteristic was also predictive of quality of life ($\beta = 0.29$, $t(145) = 2.90$, $p < 0.05$). The positive sign of the standard angular coefficient indicates that participants with better quality of life have a greater need to receive affection from people dear to them.

Generally speaking, results are consistent with the clinical markers, since increases in anxiety and symptoms of depression are associated with social or performance dysfunctions, in such a way that the individuals tend not to react when faced with criticism. This feeling is accompanied many times by a neediness for affection or aggressive reactions, although the individual is not always able to express these feelings. Moreover, both personality characteristics are associated with poor quality of life.
